# MicroRNA expression profiling during the life cycle of the silkworm (*Bombyx mori*)

**DOI:** 10.1186/1471-2164-12-284

**Published:** 2011-06-02

**Authors:** Shiping Liu, Liang Zhang, Qibin Li, Ping Zhao, Jun Duan, Daojun Cheng, Zhonghuai Xiang, Qingyou Xia

**Affiliations:** 1 The Key Sericultural Laboratory of Agricultural Ministry, College of Biotechnology, Southwest University, Chongqing 400715, PR China; 2 National Engineering Center for Beijing Biochip Technology, Life Science Parkway, Changping District, Beijing 102206, PR China; 3 Beijing Genomics Institute at Shenzhen, Shenzhen 518083, PR China; 4 Beijing Institute of Genomics, Chinese Academy of Sciences, Beijing 100000, PR China; 5 Institute of Agricultural and Life Sciences, Chongqing University, Chongqing, 400030, PR China

## Abstract

Retraction article

## Retraction

After publication of article [1], we became aware of the fact that Figures one C (let-7a and let-7a#), four B (bmo-let-7a and bmo-let-7a#) and five B (let-7a and let-7a#) were duplicated from another published article [2]. In light of these problems, the authors in consultation with the journal's Editors, have decided to retract article [1] from *BMC Genomics*. The authors are currently preparing a new manuscript clarifying the role of let-7a during the life cycle of the silkworm.
